# Evaluation of Novel Tools to Facilitate the Detection and Characterization of Leprosy Patients in China

**DOI:** 10.1155/2014/371828

**Published:** 2014-08-12

**Authors:** Yan Wen, Yuan Gang You, Lian-Chao Yuan, You Hua Yuan, Ying Zhang, Malcolm S. Duthie, Huan-Ying Li

**Affiliations:** ^1^Beijing Tropical Medicine Research Institute, Beijing Friendship Hospital, Capital Medical University, Beijing 100050, China; ^2^Department of Infectious Diseases, Huashan Hospital, Fudan University, Shanghai 200040, China; ^3^Department of Molecular Microbiology and Immunology, Bloomberg School of Public Health, Johns Hopkins University, Baltimore, MD, USA; ^4^Infectious Disease Research Institute (IDRI), 1616 Eastlake Avenue East, Seattle, WA, USA

## Abstract

Leprosy is the disabling outcome of chronic infection with *Mycobacterium leprae*. The disease often evades early detection, particularly now that fewer clinicians are able to confidently diagnose the disease following the integration of leprosy control measures within general health services in many countries. Although leprosy is officially eliminated in China, endemic regions remain in some difficult-to-reach, underdeveloped areas in Southwest China. In order to better understand the extent of *M. leprae* infection and identify new leprosy cases in a timely manner, simple tools that can detect infection and the early disease are required. In this report we evaluated the performance of antigen-specific ELISA, the NDO-LID rapid diagnostic test, and antigen-specific whole blood assays (WBA) as potential diagnostic tools. Our data support the use of antibody detection tests and WBA to facilitate the diagnosis of multibacillary and paucibacillary leprosy, respectively. These tools could be invaluable for increased, but simplified, monitoring of individuals in order to provide referrals for clinical exam and early leprosy diagnosis.

## 1. Introduction

Leprosy is the disease manifestation of infection with* Mycobacterium leprae*. The provision of free-of-charge treatment to all reported leprosy patients has led to major advances in disease control, with sharp declines in prevalence rates in the vast majority of countries over the last 20 years. China officially announced that it had reached the World Health Organization (WHO) target for leprosy elimination at a national level in 1998 [1, 2]. As in many other countries, a decentralized leprosy control program largely based on voluntary reporting or passive detection in dermatology clinics has now been adopted [1, 3, 4]. In 2010, China reported 1324 new cases of leprosy to WHO [5], with multiple factors such as age, occupation, nationality, endemicity, leprosy type, and detection methods affecting the case detection rate [6, 7].

Although leprosy patients are diagnosed throughout China, the majority of cases are found in pockets of endemicity in the ethnically diverse, mountainous, and underdeveloped southwest provinces of Yunnan, Guizhou, and Sichuan [8, 9]. The presentation of leprosy is extremely diverse, with patients exhibiting dichotomous immune responses, a wide range of bacterial burdens, and histopathological outcomes and most field programs lack the ability to thoroughly characterize the patients [10]. A simplified system suggested by WHO uses skin lesions, bacterial positivity by skin smear, and the number of involved nerves to group leprosy patients into one of two simplified categories: multibacillary (MB) and paucibacillary (PB) leprosy [11]. Even then, many clinicians cannot assuredly diagnose leprosy [3, 4, 12].

It is suspected that delayed diagnosis is quite common in China, especially because 85% of the reported cases are MB. An additional concern is that, given the rapid socioeconomic development and improved prospects in other regions, the migration of patients from highly endemic areas to nonendemic areas is increasing [8]. The development of tools that can be incorporated to facilitate diagnosis and provide a more thorough understanding of transmission and the incidence* M. leprae* infection in high endemic regions, and even throughout the country, appears to be warranted. In this report, we evaluated the performance of antigen-specific antibody detection assays (ELISA and a lateral flow-based rapid diagnostic test (RDT)) and an antigen-specific whole blood assay as potential tools for leprosy diagnosis and monitoring of* M. leprae* infection in China.

## 2. Materials and Methods

### 2.1. Study Groups

This study was approved by national and local review boards (Honghe Prefecture, Yunnan Province) and participants were included only after signing written informed consent forms. The key demographics of each group are summarized in Table 1. Patient groups comprised subjects that were either newly diagnosed and previously untreated or recently diagnosed and within the first 3 months of treatment with WHO-MDT. Both multibacillary (MB; *n* = 20) and paucibacillary (PB; *n* = 11) patients were recruited. Household contacts of both MB and PB leprosy patients were recruited as a group at elevated risk of* M. leprae* infection and development of leprosy (HHC; *n* = 42). Two additional control groups were recruited. Pulmonary tuberculosis patients under specific treatment for at least two months provided mycobacterial infection controls (TB; *n* = 11) and healthy individuals residing in the same region as the confirmed patients served as endemic controls (EC, *n* = 10).

### 2.2. Antigen-Specific Antibody Detection by Enzyme Linked Immunosorbent Assay (ELISA)

Serum antibodies recognizing each antigen were quantified by conventional ELISA, as previously described [13]. Briefly, 96-well plates (Corning, New York, USA) were coated with saturating amounts of LID-1, ND-O-BSA, or ND-O-LID before blocking with 1% BSA/ PBS/Tween-20. Serum samples diluted 1/200 in 0.1% BSA were then added in duplicate and incubated for 2 hours at room temperature. Wells were washed before incubating with a horseradish peroxidase- (HRP-) conjugated antibodies (Rockland Immunochemicals, Gilbertsville, PA) diluted in 0.1% BSA. Respectively, IgM antibodies to ND-O-BSA, IgG antibodies to LID-1, and a combination of IgM and IgG antibodies to NDO-LID were detected. After washing, plates were developed with peroxidase color substrate (Kirkegaard and Perry Laboratories, Gaithersburg, MD) and quenched by the addition of 1 N H_2_SO_4_. The optical density (OD) of each well was read at 450 nm. Results from each individual serum were expressed as the mean OD of their duplicate wells (range: 0.00–1.23). For presentation, results were then normalized by dividing by the mean OD of EC to yield a signal : noise and a threshold value for positive results of 2 was assigned.

### 2.3. Antigen-Specific Antibody Detection by NDO-LID

Serum antibodies were measured by NDO-LID rapid diagnostic test (RDT; procured from OrangeLife, Rio de Janeiro, Brazil). Briefly, NDO-LID RDT was performed by first adding undiluted serum (10 uL) into the sample well within the test cassette, followed by addition of a running buffer (100 uL). Sample migrated through the cassette such that interactions with the test and/or control lines could be revealed as red colored lines within the reading window. Tests were valid if the control line was revealed. A positive result was defined by the revelation of the test line. Visual results were interpreted after 20 minutes by two independent readers and scored subjectively as ±/+/++/+++), with faint (±) or no test line development considered as a negative result.

### 2.4. Antigen-Specific T Cell Responses

Antigen-specific cell-mediated immune responses were determined using whole blood assay (WBA) and subsequent measurement of IFN*γ*. WBA was conducted by adding 450 uL/well undiluted venous heparinized whole blood to 48-well tissue culture plates (Corning Costar, Tewksbury, MA) with protein added to provide a final volume of 500 uL per well. Blood was incubated with 10 ug/mL recombinant protein (single antigens ML2028 and ML2044, and fusion protein LID-1, which combines ML0405 and ML2331), PBS (negative control), or 75 ug/mL PHA (positive control; Sigma Aldrich, St. Louis, MO). Following incubation at 37°C, without 5% CO_2_ for 24 hours_,_ plasma was carefully collected and IFN*γ* content was measured by ELISA according to manufacturer's instructions (Haikou VTI Biological Institute, China). A threshold for positive responses was arbitrarily selected at 50 pg/mL.

### 2.5. Statistics

Statistical significance was assessed using unpaired *t*-test for comparison between two groups. Results were considered statistically significant when *P* values <0.05 were obtained. The concordance between results was determined by agreement and kappa values (*κ*) with 95% CI and SD values were calculated (Statistical Package for the Social Sciences (SPSS) version 13.0).

## 3. Results

### 3.1. Utility of Conventional ELISA for the Confirmation of Leprosy

**Figure 1:**
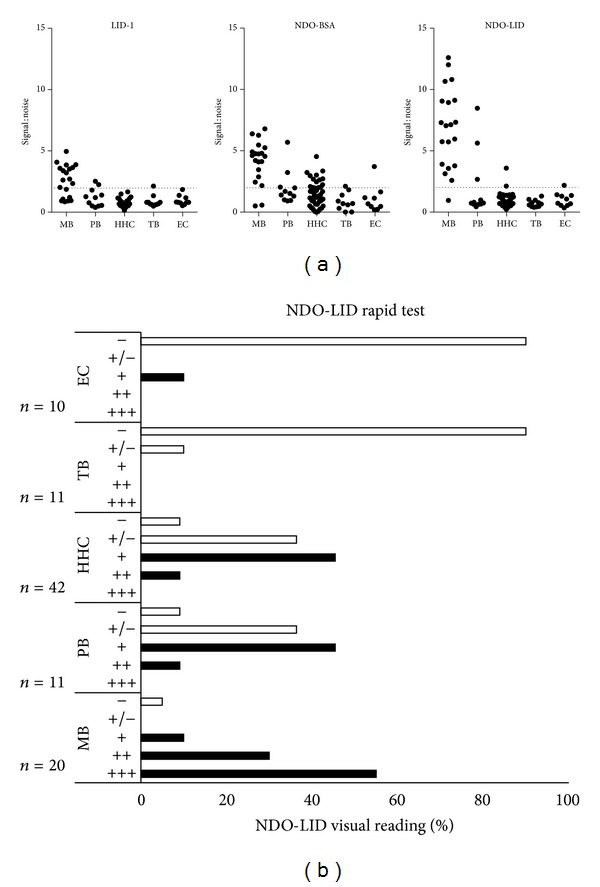
Antibody responses of leprosy patients. Sera from leprosy patients (MB = 20 and PB = 11), healthy household contacts of confirmed patients (HHC = 42), pulmonary tuberculosis patients (TB = 11), and uninfected controls (EC = 10) were assessed. Responses were measured by ELISA against (a) LID-1, ND-O-BSA, and NDO-LID. Results from each individual serum sample were normalized against the mean OD obtained from EC to allow presentation as signal : noise and are distinguished by an individual marker. The dashed horizontal line represents the threshold for positive results (2 × mean EC). Within the PB classification, 2/6 BT and 2/5 TT patients were positive for LID-1; 2/6 BT and 2/5 TT patients were positive for NDO-LID; and 3/6 BT and 2/5 TT patient were positive for NDO-BSA. In (b) NDO-LID tests were developed and scored by visual interpretation. Results for each group are summarized into scoring category, and the percentage of samples that were unambiguously positive (≥+) is listed to the right. Within the PB classification, 3/6 BT and 4/5 TT patients were positive.

It is well established that MB patients typically have high antibody titers against* M. leprae* antigens while PB patients do not [14]. To determine the antibody responses against various* M. leprae* antigens of MB, PB, HHC, TB, and EC residing in the highly leprosy endemic Honghe Prefecture of Yunnan Province, serum antibody levels were measured by ELISA (Figure 1(a) and Table 2). While 90% (18/20) of MB and 45.5% (5/11) of PB sera demonstrated positive responses against PGL-I by NDO-BSA ELISA, antibodies against LID-1 were detected in 65% (13/20) of MB and 36.4% (4/11) of PB sera, respectively. When the same samples were evaluated against ND-O-LID, the single conjugated product of these antigens, 95% (19/20) of MB sera and 36.4% (4/11) of PB sera were positive in ELISA. These data indicate the utility of conventional antigen-specific ELISA in supporting clinical diagnosis of leprosy among Chinese patients.

### 3.2. Performance of NDO-LID Rapid Tests

We recently developed a lateral flow test based on the ND-O-LID conjugate that can provide results within 20 minutes of sample addition [15, 16]. Serum samples were therefore also evaluated in this RDT platform (Figure 1(b)). As expected, the confirmation was achieved in 95% of MB patients by the rapid test and a high degree of agreement was observed with LID-1, NDO-BSA, and ND-O-LID ELISA (Table 3). Interestingly, the confirmation of PB leprosy was increased to 63.6%, such that the NDO-LID RDT demonstrated the highest rate of detection of PB leprosy patients while concomitantly yielding the lowest rate of cross-reaction with control samples (TB and EC) (Figure 1(b)). These data indicate an improved discriminating capacity of the lateral flow format over conventional ELISA for detecting the disease.

### 3.3. Antigen-Specific Responses in Whole Blood Assay (WBA)

**Figure 2:**
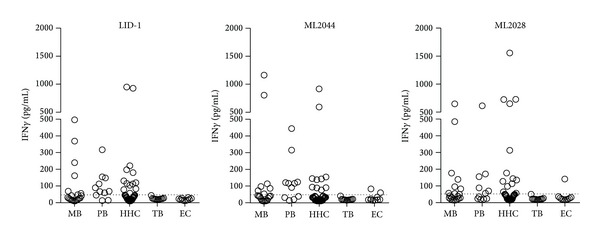
Antigen recall responses of leprosy patients. Venous heparinized whole blood from PB, MB, HHC, TB, and EC was incubated with various antigens for 24 hours; then plasma was collected, and IFN*γ* content was measured by ELISA. Results from each individual WBA are distinguished by an individual marker. The dashed horizontal line represents the cutoff (50 pg/mL) for positive results.

Despite the relative absence of antibody responses, PB patients and HHC do typically demonstrate cellular recall responses to* M. leprae* antigens [14]. To provide a fuller characterization of our study population, antigen-specific cellular responses were therefore assessed with MB, PB, HHC, TB, and EC samples (Figure 2). A small proportion of MB patients responded by secreting IFN*γ* upon incubation with the antigens in WBA, with positive responses stimulated in 35.0%, 35.0%, and 40.0% by incubation with the LID-1, ML2044, and ML2028 proteins, respectively. As anticipated, higher rates of positive responses were detected following incubation of PB patient blood with LID-1 (72.7%), ML2044 (63.6%), and ML2028 (54.5%). Similarly, positive responses were detected following incubation of HHC blood with LID-1 (31.0%), ML2044 (23.8%), and ML2028 (31.0%). Responses were not elicited by these proteins upon incubation with blood from TB patients (0/11), and only weakly by ML2028 (1/10) and ML2044 (2/10) upon incubation with EC blood. These data indicate the highly specific nature of responses in patients or* M. leprae*-exposed individuals. Taken together, our data indicate that LID-1 WBA were highly specific and had higher sensitivity for PB leprosy than the other antigens evaluated.

## 4. Discussion

Advances in the provision of treatment during the 1980s and 1990s massively reduced the worldwide suffering caused by leprosy, with an ultimate goal of elimination (defined as <1 case per 10,000 individuals) by 2000. The stalled decreases in both global prevalence and new case detection rates over the last decade, however, indicate that additional measures are likely required. Indeed, more recent evidence suggests that the integration of many control programs into general health services may have been premature and could be causing delays in the diagnosis and treatment of leprosy.

Many clinicians, even in leprosy endemic regions, are not capable of confidently diagnosing leprosy [3, 4, 12]. This is not entirely unexpected because leprosy presents with a spectrum of* M. leprae*-specific immune phenotypes ranging from being almost entirely biased to antibody responses to almost entirely biased to cell-mediated responses, which are associated with the extremely varying bacterial burdens and histopathological outcomes observed in patients [10]. At the extreme pole of the MB presentation, lepromatous (LL) disease is characterized by weak or absent* M. leprae*-specific cell-mediated immunity and high antibody titers such that bacillary replication and dissemination lead to high bacterial indices (BI). Consistent with previous reports, our data support the use of antibody detection assays at some point in the diagnostic algorithm. Responses were readily detected against both phenolic glycolipid- (PGL-) I (NDO-BSA) and LID-1 in sera from MB patients, with the single conjugated product of these antigens, NDO-LID, delivering enhanced performance at a sensitivity of 95% for MB leprosy and specificity of 95.2% (1 positive from 21 combined EC and TB). ELISA is consistently performed in reference laboratories and could be used to either confirm diagnosis or facilitate referral for expert examination for individuals suspected of having leprosy. We have recently codeveloped Leprosy Detect fast ELISA that further simplifies and expedites this process [13]. The NDO-LID RDT evaluated is suited to point-of-care application and could be used as a primary detection method to, at least, serve as an access point for clinical exam or more thorough immunological or bacteriological assessment.

At the other extreme of the leprosy spectrum, tuberculoid (TT) patients present strong* M. leprae*-specific cell-mediated immunity and low or absent antibody production, such that patients present with low BI. PB patients are diagnosed based on symptoms and histopathology because BI are negative. The Mitsuda skin test (intradermal injection of heat-killed* M. leprae* then measurement of a granulomatous reaction 21–30 days later) has been used in limited settings to assess cell-mediated immunity. Practical limitations, coupled with the observation that the Mitsuda test can be positive in the majority of individuals even in leprosy nonendemic regions, have precluded its use for diagnosis [17, 18]. To address the diagnostic deficit that exists for PB patients and consistent with the approach used to develop tests for TB diagnosis [19–21], a small number of research groups have been investigating* M. leprae* antigen-specific T cell responses. Our own work has identified multiple antigens that elicit IFN*γ* upon incubation with blood from PB patients in Brazil [22–25]. The data presented here extend these activities to the Chinese leprosy population but, consistent with recent data, indicate that additional antigens or markers are needed to enhance the sensitivity of the response [26]. Our data indicate that TB patients and EC did not respond to* M. leprae* antigen stimulation whereas a large proportion of PB patients, and even some MB patients, did. As with other studies, HHC also responded to the* M. leprae* antigen stimulation. Thus, antigen responsiveness likely reveals exposure to or potential asymptomatic infection with* M. leprae*. Although the WBA could therefore be used in conjunction with clinical suspicion to support PB diagnosis, the broader utility would appear to be as a screening tool to measure* M. leprae* transmission.

Despite the official elimination of leprosy in China, endemic pockets remain in some provinces [27, 28]. In addition, the high proportion of MB cases that are observed suggests that delays in diagnosis may be common. Such delays not only increase the likelihood of permanent nerve damage in patients but also permit transmission of* M. leprae* to continue [29–31]. The use of practical tools such as those outlined here could facilitate diagnosis or, at a minimum, instruct referral to experts and could potentially fill the current deficit in clinical awareness of leprosy. Our data also support the use of the tools investigated to study and quantify leprosy transmission within such regions.

## Figures and Tables

**Table 1:** Study group demographics.

Group	*n*	m/f	Mean age (range)	Ridley-Jopling classification	Bacterial index (BI)	
					Skin	Pathology
MB	20	13/7	39.5 (20–86)	1 LL; 18 BL; 1 BB	2.7 (0–4.5)^1^	3.4 (0–5+)^2^
PB	11	8/3	44.9 (27–79)	5 TT; 6 BT	0.1 (0–0.5)^3^	0.4 (0–3.5+)^3^
HHC	42	16/26	28.5 (4–82)	na	na	
TB	11	8/3	39.5 (23–72)	na	na	
EC	10	7/3	42.2 (35–48)	na	na	

^1^Not reported for 4 patients.

^2^Not reported for 3 patients.

^3^Not reported for 2 patients.

na, not applicable.

**Table 2:** Summarized results of three tests evaluated.

Group	*n*	Rapid test	ELISA^1^			WBA^2^		
		NDO-LID	LID-1	NDO-BSA	ND-O-LID	LID-1	ML2044	ML2028
Confirmed patients								
MB	20	95% (19)	65% (13)	90% (18)	95% (19)	35% (7)	35% (7)	40% (8)
PB	11	63.6% (7)	36.4% (4)	45.5% (5)	36.4% (4)	72.7% (8)	63.6% (7)	54.5% (6)
At risk								
HHC	42	21.4% (9)	0% (0)	35.7% (15)	2.4% (1)	31.0% (13)	23.8% (10)	31.0% (13)
Controls								
TB	11	0% (0)	9.1% (1)	9.1% (1)	0% (0)	0% (0)	0% (0)	0% (0)
EC	10	10% (1)	0% (0)	10% (1)	10% (1)	0% (0)	20% (2)	10% (1)
Combined	21	4.8% (1)	4.8% (1)	9.5% (2)	4.8% (1)	0% (0)	9.5% (2)	4.8% (1)

^1^ELISA was determined as positive if mean OD of duplicate wells was >2 × EC mean OD.

^2^WBA were determined as positive if IFN*γ* concentration was >50 pg/mL.

**Table 3:** Analyses for agreement of results by the various test formats.

Patient classification			Kappa test	
			Kappa	*P* value
MB	NDO-LID versus ELISA^1^ with	LID-1	—	—
		NDO-BSA	0.643	<0.01
		ND-O-LID	1.000	<0.01

PB		LID-1	0.068	ns^3^
		NDO-BSA	0.154	ns
		ND-O-LID	0.029	ns

MB	NDO-LID versus WBA^2^ with	LID-1	0.055	ns
		ML2044	0.055	ns
		ML2028	0.102	ns

PB		LID-1	0.377	ns
		ML2044	0.607	<0.05
		ML2028	0.441	ns

^1^ELISA was determined as positive if mean OD of duplicate wells was >2 × EC mean OD.

^2^WBA were determined as positive if IFN*γ* concentration was >50 pg/mL.

^3^ns, not significant.
